# Complete Genome Sequences of Two *Escherichia coli* O145:H28 Outbreak Strains of Food Origin

**DOI:** 10.1128/genomeA.00482-14

**Published:** 2014-05-22

**Authors:** Kerry K. Cooper, Robert E. Mandrell, Jacqueline W. Louie, Jonas Korlach, Tyson A. Clark, Craig T. Parker, Steven Huynh, Patrick S. G. Chain, Sanaa Ahmed, Michelle Qiu Carter

**Affiliations:** a Produce Safety and Microbiology Research Unit, Western Regional Research Center, Agricultural Research Service, U.S. Department of Agriculture, Albany, California, USA; b Pacific Biosciences, Menlo Park, California, USA; c Genome Sciences, Bioscience Division, Los Alamos National Laboratory, Los Alamos, New Mexico, USA

## Abstract

*Escherichia coli* O145:H28 strain RM12581 was isolated from bagged romaine lettuce during a 2010 U.S. lettuce-associated outbreak. *E. coli* O145:H28 strain RM12761 was isolated from ice cream during a 2007 ice cream-associated outbreak in Belgium. Here we report the complete genome sequences and annotation of both strains.

## GENOME ANNOUNCEMENT

Shiga toxin-producing *Escherichia coli* O145 was identified as a cause of hemolytic uremic syndrome (HUS) at least two decades ago (1–3), and now is recognized as one of the six non-O157 serotypes that are most frequently associated with human disease in the United States (4, 5). Food-borne outbreaks of O145 infection have been reported worldwide (6–9). *E. coli* O145:H28 strain RM12581 was isolated from bagged romaine lettuce during a multistate outbreak of O145 infections associated with romaine lettuce consumption in April and May 2010 in the United States (9). Strain RM12761 was isolated from ice cream during an outbreak of O145 and O26 infections among ice cream consumers in Belgium in 2007 (7, 10). We reported previously the complete genome sequence of a clinical isolate linked to each of the two outbreaks (11).

Whole-genome sequencing (WGS) and 8-kb insert paired-end (PE) 454 sequencing libraries were prepared and sequenced on an FLX Genome Sequencer (Roche) using Titanium chemistry. Illumina library preparation and sequencing (101 bp PE) were run at Ambry Genetics (Aliso Viejo, CA) on an HiSeq2000 sequencer. PacBio libraries for circular consensus sequence (CCS) reads were prepared and PacBio SMRT sequencing was run on a PacBio RS instrument using C2 chemistry. Draft genomes were assembled using Newbler (v2.3) for 454 reads and VELVET (v1.0.13) for Illumina PE reads as described previously (11). A total of 13 scaffolds composed of 158 contigs and 2 scaffolds composed of 6 contigs were generated for RM12581 and RM12761, respectively, after gap closure and misassembly correction using GapResolution and dupFinisher (12, 13). PacBio CCS reads were then aligned to the contigs using Geneious (v5.1) software (14), and the remaining gaps were manually closed *in silico*. Annotation was carried out using RAST (http://rast.nmpdr.org) (15).

The *E. coli* RM12581 genome is composed of a 5,585,611-bp chromosome and two large plasmids, pO145-12581 (81,120 bp) and pRM12581 (64,562 bp), and contains 5,769 coding DNA sequences (CDSs), 22 rRNAs, and 104 tRNAs. The *E. coli* RM12761 genome is composed of a 5,402,281-bp chromosome and two large plasmids, pO145-12761 (98,067 bp) and pRM12761 (58,666 bp), and contains 5,493 CDSs, 22 rRNAs, and 98 tRNAs. Single nucleotide polymorphisms (SNPs) between the strains of food and clinical origins linked to the same outbreak (RM12581 and RM13514; RM12761 and RM13516) were identified using breseq (v0.23) (16, 17). A total of 18 SNPs were identified in non-repeat regions for the 2007 Belgian ice cream-associated outbreak strains, of which 11 are in intergenic regions and seven are in coding regions (4 synonymous SNPs, 2 nonsense mutations, and 1 nonsynonymous mutation). The two CDSs with a nonsense mutation encode an endonuclease (ECRM13516_0820) and a hypothetical protein (ECRM13516_2566). The nonsynonymous mutation was detected in the gene encoding a valyl-tRNA synthetase (ECRM13516_5090). In contrast, no SNPs were identified in non-repeat regions between the two strains linked to the 2010 U.S. lettuce-associated outbreak. Nearly identical methylomes were detected between the two *E. coli* O145 strains of food and clinical origins that were linked to the same outbreak (11).

### Nucleotide sequence accession numbers.

The *E. coli* O145:H28 strain RM12581 and RM12761 genome sequences were deposited at DDBJ/EMBL/GenBank under the accession no. CP007136 to CP007138 and CP007133 to CP007135, respectively.

## References

[1] Karmali MA Petric M Lim C Fleming PC Arbus GS Lior H 1985 The association between idiopathic hemolytic uremic syndrome and infection by verotoxin-producing *Escherichia coli*. J. Infect. Dis. 151:775–782. 10.1093/infdis/151.5.775 3886804

[2] Chart H van der Kar NC Tolboom JJ Monnens LM Rowe B 1993 Serological detection of verocytotoxin-producing *Escherichia coli* in patients with haemolytic uraemic syndrome in Western Europe. Eur. J. Clin. Microbiol. Infect. Dis. 12:707–709. 10.1007/BF02009386 7694851

[3] Beutin L Aleksic S Zimmermann S Gleier K 1994 Virulence factors and phenotypical traits of verotoxigenic strains of *Escherichia coli* isolated from human patients in Germany. Med. Microbiol. Immunol. 183:13–21 8202027 10.1007/BF00193627

[4] Mathusa EC Chen Y Enache E Hontz L 2010 Non-O157 Shiga toxin-producing *Escherichia coli* in foods. J. Food Prot. 73:1721–1736 20828483 10.4315/0362-028x-73.9.1721

[5] Brooks JT Sowers EG Wells JG Greene KD Griffin PM Hoekstra RM Strockbine NA 2005 Non-O157 Shiga toxin-producing *Escherichia coli* infections in the United States, 1983–2002. J. Infect. Dis. 192:1422–1429. 10.1086/466536 16170761

[6] Heinikainen S Pohjanvirta T Eklund M Siitonen A Pelkonen S 2007 Tracing shigatoxigenic *Escherichia coli* O103, O145, and O174 infections from farm residents to cattle. J. Clin. Microbiol. 45:3817–3820. 10.1128/JCM.00198-07 17804658 PMC2168472

[7] De Schrijver K Buvens G Posse B Van den Branden D Oosterlynck O De Zutter L Eilers K Pierard D Dierick K Van Damme-Lombaerts R Lauwers C Jacobs R 2008 Outbreak of verocytotoxin-producing *E. coli* O145 and O26 infections associated with the consumption of ice cream produced at a farm, Belgium, 2007. Euro Surveill. 13:pii=8041 http://www.eurosurveillance.org/ViewArticle.aspx?ArticleId=8041 10.2807/ese.13.07.08041-en 18445416

[8] Much P Pichler J Kasper SS Allerberger F 2009 Foodborne outbreaks, Austria 2007. Wien. Klin. Wochenschr. 121:77–85. 10.1007/s00508-008-1125-z 19280130

[9] Taylor EV Nguyen TA Machesky KD Koch E Sotir MJ Bohm SR Folster JP Bokanyi R Kupper A Bidol SA Emanuel A Arends KD Johnson SA Dunn J Stroika S Patel MK Williams I 2013 Multistate outbreak of *Escherichia coli* O145 infections associated with romaine lettuce consumption, 2010. J. Food Prot. 76:939–944. 10.4315/0362-028X.JFP-12-503 23726187

[10] Buvens G Posse B De Schrijver K De Zutter L Lauwers S Pierard D 2011 Virulence profiling and quantification of verocytotoxin-producing *Escherichia coli* O145:H28 and O26:H11 isolated during an ice cream-related hemolytic uremic syndrome outbreak. Foodborne Pathog. Dis. 8:421–426. 10.1089/fpd.2010.0693 21166561

[11] Cooper KK Mandrell RE Louie JW Korlach J Clark TA Parker CT Huynh S Chain PS Ahmed S Carter MQ 2014 Comparative genomics of enterohemorrhagic *Escherichia coli* O145:H28 demonstrates a common evolutionary lineage with *Escherichia coli*. BMC Genomics 15:17. 10.1186/1471-2164-15-17 24410921 PMC3893438

[12] Ahmed SA Awosika J Baldwin C Bishop-Lilly KA Biswas B Broomall S Chain PSG Chertkov O Chokoshvili O Coyne S Davenport K Detter JC Dorman W Erkkila TH Folster JP Frey KG George M Gleasner C Henry M Hill KK Hubbard K Insalaco J Johnson S Kitzmiller A Krepps M Lo CC Luu T McNew LA Minogue T Munk CA Osborne B Patel M Reitenga KG Rosenzweig CN Shea A Shen X Strockbine N Tarr C Teshima H van Gieson E Verratti K Wolcott M Xie G Sozhamannan S Gibbons HS Threat Characterization Consortium 2012 Genomic comparison of *Escherichia coli* O104:H4 isolates from 2009 and 2011 reveals plasmid, and prophage heterogeneity, including Shiga toxin encoding phage stx2. PLoS One 7:e48228. 10.1371/journal.pone.0048228 23133618 PMC3486847

[13] Han C Chain P 2006 Finishing repetitive regions automatically with Dupfinisher, p 142–147 Arabnia HR Valafar H (ed), Proceedings of the 2006 International Conference on Bioinformatics & Computational Biology, BIOCOMP’06, Las Vegas, NV

[14] Kearse M Moir R Wilson A Stones-Havas S Cheung M Sturrock S Buxton S Cooper A Markowitz S Duran C Thierer T Ashton B Meintjes P Drummond A 2012 Geneious basic: an integrated and extendable desktop software platform for the organization and analysis of sequence data. Bioinformatics 28:1647–1649. 10.1093/bioinformatics/bts199 22543367 PMC3371832

[15] Aziz RK Bartels D Best AA DeJongh M Disz T Edwards RA Formsma K Gerdes S Glass EM Kubal M Meyer F Olsen GJ Olson R Osterman AL Overbeek RA McNeil LK Paarmann D Paczian T Parrello B Pusch GD Reich C Stevens R Vassieva O Vonstein V Wilke A Zagnitko O 2008 The RAST server: Rapid Annotations using Subsystems Technology. BMC Genomics 9:75. 10.1186/1471-2164-9-75 18261238 PMC2265698

[16] Barrick JE Lenski RE 2009 Genome-wide mutational diversity in an evolving population of *Escherichia coli*. Cold Spring Harb. Symp. Quant. Biol. 74:119–129. 10.1101/sqb.2009.74.018 19776167 PMC2890043

[17] Barrick JE Yu DS Yoon SH Jeong H Oh TK Schneider D Lenski RE Kim JF 2009 Genome evolution and adaptation in a long-term experiment with *Escherichia coli*. Nature 461:1243–1247. 10.1038/nature08480 19838166

